# Calming the Storm: Natural Language Processing Applied to Pulmonary Embolism Research

**DOI:** 10.1016/j.jacadv.2025.101957

**Published:** 2025-07-23

**Authors:** Kevin O'Gallagher, Jack Wu, Richard JB. Dobson

**Affiliations:** a School of Cardiovascular and Metabolic Medicine & Sciences, British Heart Foundation Centre of Research Excellence, King's College London, London, United Kingdom; b King's College Hospital NHS Foundation Trust, London, United Kingdom; c Department of Biostatistics and Health Informatics, Institute of Psychiatry, Psychology and Neuroscience, King's College London, London, United Kingdom

**Keywords:** electronic health records, natural language processing, pulmonary embolism

**Figure:**
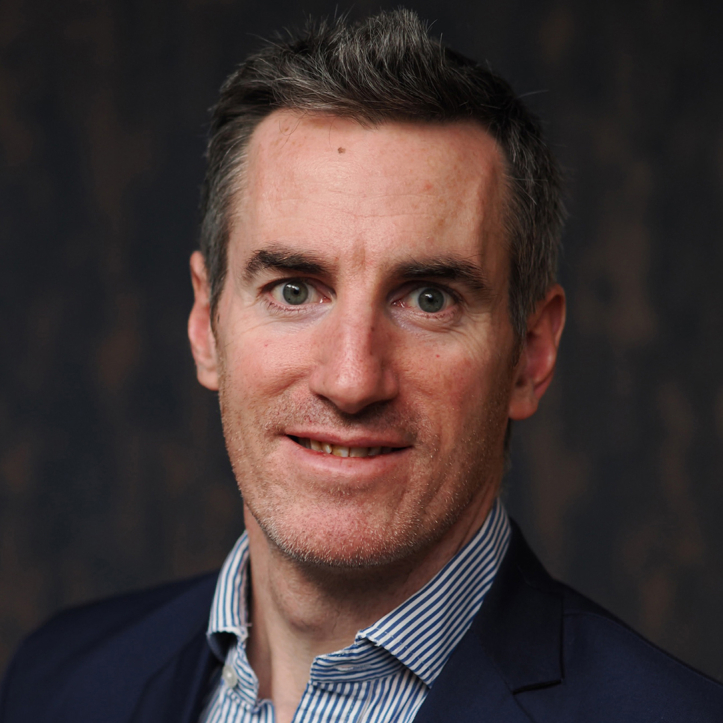

Pulmonary embolism (PE) is an important, life-threatening cardiovascular condition. As the authors of the accompanying article note, PE affects 600,000 patients in the United States every year, with significant rates of morbidity and mortality.^1^

PE is difficult to diagnose yet can be fatal if missed. Initial testing can be highly sensitive, yet frustratingly nonspecific. There is an expanding range of treatment options, but each modality has its own risks and the optimal management algorithm for individual patients may not always be clearly defined.

The current paradigm for the diagnosis of PE involves incorporating a number of factors to define the likelihood of a diagnosis, which then guides the need for cross-sectional imaging. Although blood test such as D-dimer can identify thromboembolism, they lack specificity and therefore inappropriate use can lead to unnecessary use of computed tomography (CT) with the attendant ionizing radiation.^2^

PE therefore falls into a similar category of a “perfect storm” as other acute cardiovascular conditions such as aortic dissection: they can be extremely difficult to diagnose yet at the same time a failure to make an accurate diagnosis can result in death.

As the authors argue, PE research stands to benefit from multicenter data collection to enable construction of large-scale registries, allowing analysis for hypothesis-generating research to guide clinical trials in the field, with additional benefits such as benchmarking for best practice.

In their retrospective study in this issue of *JACC: Advances*, Hassan et al^3^ leveraged electronic health records (EHRs) from the Mass General Brigham (MGB) system, developing a two-stage hybrid natural language processing (NLP) pipeline for PE identification from radiology reports. In the first stage, a machine learning (ML)-based NLP model was trained and tested on 1,040 CT pulmonary angiography reports from a single tertiary care hospital in MGB system, with 80:20 splitting of training and testing cohorts and stratified sampling. Text preprocessing included stop-words removal, tokenization, and term frequency–inverse document frequency (TF-IDF) vectorization with 5,000 features. It was essentially implementing a classic bag-of-words model that represents each report as a sparse vector of TF-IDF term weights. Four classifiers (logistic regression, random forest, support vector machine [SVM], and naive Bayes) were trained using five-fold cross-validation and grid-search hyperparameter tuning, with class-weight adjustments to handle imbalance data. The fine-tuned SVM model, selected for its optimal balance of sensitivity and specificity, was then deployed on a larger set of 49,611 CT pulmonary angiography reports extracted from the remaining hospitals in the MGB system.

In the second stage, to refine the model's performance, a rule-based NLP component using expert-driven regular expressions was iteratively applied to correct for false positives and false negatives in the larger data set. Blinded manual expert review of 500 randomly sampled reports provided gold standard labels to validate model predictions. Performance was assessed using standard diagnostic metrics (accuracy, sensitivity, specificity, positive predictive value, negative predictive value, and area under the receiver operating characteristic curve). This hybrid approach benefits from the efficiency of ML for pattern recognition and the specificity of manually curated rule-based systems, enabling accurate classification of PE cases with modest computational requirements.

A key feature of the work is the hybrid architecture which pairs ML's pattern recognition with simple rule-based regular expression matching to maximize performance while minimizing annotation efforts. Scaling from 1,040 reports from a single hospital to 49,611 reports across multiple hospitals showcased robust generalizability, with postrefinement accuracy using rule-based regular expression matching rising from 85% to 94.8%, sensitivity to 96.4%, and specificity to 93.2%. The use of classic TF-IDF bag-of-words and SVM models ensures rapid training and deployment in environments without graphical processing units, which provides broad reproducibility in hospitals with modest computational resources. The manually curated rule-based regular expressions result in high transparency which allows straightforward customization by domain experts during external deployments. This combination of small data requirements, computational simplicity, and adaptability enables the pipeline for rapid translation into real-time clinical decision support systems.

Despite its advantages, the pipeline has some limitations, whose relevance is not restrained to the field of PE. First, all data were extracted from one integrated health system (the MGB system) which means that different EHR architectures or radiology reports in other settings may degrade performance and therefore require external validation in diverse geographic regions. Moreover, successful adaptation in other settings will require substantial local customization of the rule-based components, relying heavily on manual expert input to define and maintain context-specific terminology and patterns.

Second, the training cohort was predominantly White and insured, so model equity across underrepresented racial, ethnic, and socioeconomic subgroups remains unknown as no subgroup analyses were reported. There is a significant body of work demonstrating that socioeconomic and race and ethnicity-based factors heavily influence cardiovascular care and outcomes across a range of cardiovascular conditions. However, future optimization of the pipeline to ensure model equity across underrepresented groups is possible and should be encouraged.

Finally, while manual review ensures a reliable gold standard, it is labor-intensive and subject to inter-rater variability. Semisupervised or active-learning approaches could help to ease this problem. To address these gaps, a multisystem validation, equity and fairness analysis, contextualized embeddings (eg, ClinicalBERT), spelling-correction preprocessing, and suitable large language models (LLMs) integration would further strengthen generalizability and equity of the pipeline.

## Future perspectives

Traditional NLP pipelines are considered a good strategy in health care because they require far fewer labeled examples, based on transparent decision logic, integrate easily into existing EHR infrastructures, and avoid the unpredictable “black box” behaviors like hallucinations from LLMs.^4^ They also provide explainable feature importance, facilitate regulatory compliance, and support rapid deployment without high investment in compute costs.

Looking forward, this NLP framework can be extended to extract richer clinical phenotypes, for examples, clot burden, location, and chronicity from radiology reports. It could flag suspected PE cases at the point of interpretation. Integrating structured data (eg, vital signs, laboratory results) with NLP outputs could enable comprehensive risk stratification. Active learning and semisupervised ML approaches may further reduce annotation needs, also incorporating domain-adapted LLMs and appropriate prompt engineering could capture detailed contextual information and enhance detection of rare cases from the EHRs. The NLP framework could also be applied to compile cases of important yet understudies sequelae of PE such as post-PE syndrome and recurrent PE.^5^

More generally, the potential applications of NLP are not restricted to simply compiling large-scale retrospective data. For example, an NLP strategy could be employed to address the issue of representativeness of PE clinical trial populations. Many randomized controlled trials fail to reflect the demographic and clinical diversity of real-world patient populations, limiting the generalizability of their findings. In cardiovascular research, trial participants are often younger, more likely to be male, and differ in clinical characteristics compared to the broader patient base.^6^ NLP applied to EHR data would enable trialists to ensure that their recruited study matches the demographic and clinical characteristics of the underlying disease population at each participating trial site, thus ensuring the “generalizability” of the clinical trial results. It can also be envisaged that NLP could be used in a prospective fashion to deploy prompts for patients and inform adaptive recruitment strategies.

In summary, Hassan et al^3^ should be congratulated for their application of AI-based technology to facilitate important research in the field of PE.

## Funding support and author disclosures

Dr O'Gallagher is supported by an Medical Research Council Clinician Scientist Fellowship (MR/Y001311/1). Prof Dobson is the Co-Founder of CogStack. All other authors have reported that they have no relationships relevant to the contents of this paper to disclose.
